# Factors Influencing Nursing Students’ Immersive Virtual Reality Media Technology-Based Learning

**DOI:** 10.3390/s21238088

**Published:** 2021-12-03

**Authors:** Young-Ju Kim, Sung-Yun Ahn

**Affiliations:** 1 Department of Nursing, Daejeon Health Institute of Technology, Daejeon 34504, Korea; yjkim@hit.ac.kr; 2 Department of Nursing, Pai Chai University, Daejeon 35345, Korea

**Keywords:** virtual reality, learning, immersive media technology, teaching method, students

## Abstract

Background/objectives: This study aims to identify the effects of cognitive and emotional variables related to immersive virtual reality media technology on learning for nursing students. Methods/Statistical analysis: The subjects of this study were 121 nursing students from a university in area D. After experiential learning with virtual reality from 6–8 June 2019, data was collected through questionnaires. For virtual reality learning, VIVE’s hTC VIVE ECO CE model was used. The collected data was analyzed using the IBM SPSS 26.0 program. Multiple Regression Analysis was used to analyze the factors influencing the subject’s virtual reality learning effects. Findings: The learning effects of the virtual reality medium had a statistically significant positive correlation with the virtual reality technology recognition, sensory immersion, realism, learning satisfaction, learning necessity, and continuous use intention (*p* < 0.001) scores. In personality traits, only Openness, Extraversion (*p* < 0.01), and Conscientiousness (*p* < 0.05) had a statistically significant positive correlation. As a result of regression analysis, the explanatory power of the learning effect of the virtual reality medium was 63.9% (F = 53.61, *p* < 0.001), with learning satisfaction, sensory immersion, continuous use intention, and Extraversion being significant influencing factors (*p* < 0.05). Improvements/Applications: This study is meaningful in the sense that it provided strategic implications for the teaching and learning method of virtual reality technology-based learning by considering the insights necessary to develop a learning program using virtual reality technology, according to the characteristics of virtual reality technology, and the learner’s cognitive and psychological variables.

## 1. Research Needs

With the development of educational technology, more and more innovative learning tools have been provided in recent years [1]. In particular, the field of nursing practice is regarded as a very important process for learners to develop the ability to apply skills and expertise. Therefore, it is very important to identify which learning methods are the most effective for proficiency development to ensure the competence of graduates who have completed the nursing program as practitioners.

Among the practical competencies to be acquired, the most difficult to learn are those that occur rarely and are not easy to experience in practice, but which are also complicated to perform directly due to certain practical experiences that are essential for the safety of patients and students. To compensate for this, efforts have been made to supplement such practice through equipment that incorporates cutting-edge technology, such as using a high-fidelity simulator. As a result, practice using the high-fidelity simulator was evaluated as having advantages regarding ethical issues and increasing confidence in nursing performance. However, the high-fidelity simulator has high-cost problems, such as the purchasing costs and management costs, as well as the costs of maintaining the equipment and securing an installation space. There are also limitations in learner input [2] and limitations in reproducing reality in the practice contents.

Recently, with the advent of the 4th Industrial Revolution and the development of IT technology, attempts to introduce virtual reality simulation (VRS), which can overcome the limitations of education based on simulator equipment, has been put into practice [3] in various academic fields. At the same time, because of the spread of COVID-19, the face-to-face practice has become difficult for nursing departments, and the use of virtual reality media is attracting attention as an effective alternative practice method.

Virtual reality is an imaging technology that provides a realistic environment to the subject by providing real-time feedback in a virtual environment [4]. Since practice in a virtual reality medium allows for practice on a virtual patient existing in cyberspace, the advantages of the existing high-fidelity simulator, such as ethical issues about patient safety, are maintained and the disadvantages are resolved. In other words, compared to high-fidelity simulators, which can be organized only with a small number of learners and limited in operation [2], virtual reality media can simulate clinical situations with a high sense of reality [5] without spatial and temporal constraints, so effective learning can be expected through a high level of immersion.

Accordingly, various studies have been conducted, such as an integrative review study centered on experimental research that was conducted from 2016 to 2019 to confirm the effect of nursing education using VR, and performance, self-confidence, and learning satisfaction were significantly improved [6]. In addition, a recent experimental study showed that the experimental group that performed VR simulations to provide safe clinical expiration of neonatal intensive care units with limited access to nursing students had a higher high-risk neural infrastructure self-efficacy and learner status than the control group [7].

Although many studies related to virtual reality media have been conducted for nursing students, looking at the contents, it can be observed that most of them are focused on the development of virtual reality educational content then the ability to cope with multiple trauma situations [8] or programs [9]. As a result of previous research that was conducted to confirm the effectiveness of virtual reality simulation education through an integrative analysis on quantitative and qualitative works, it was found that readiness for the use of a virtual reality device, a mastering of the platform, and an interesting scenario were necessary [10]. This shows that VR-applied game-like hands-on education has the characteristics of a high level of immersion [11]. In addition, the biggest characteristic of virtual reality media is that learners learn with a constructivist approach through VR [5], which is that it presupposes self-directed learner-centered education [12] unlike the existing teacher-centered learning method.

Because VR is a self-directed, learner-centered educational method with a highly immersive medium, it can be expected that the cognitive or emotional variables of the learner-centered virtual reality medium, including personality characteristics of the learner, will have the greatest influence on the overall learning effect. Therefore, it is necessary to confirm how education through VR affects the cognitive and emotional variables of learning. However, only when learning methods through new media are introduced, such as distance learning with media [13,14], augmented reality-based learning [15], smart device application learning [16], and the learners’ perception of media technology and virtual reality, studies have been conducted to confirm the influence of cognitive and emotional variables on the medium itself, such as media characteristics, virtual reality learning satisfaction, learning demand, and intention of continued use. Regarding nursing education through VR, insufficient studies were confirming the cognitive and emotional effects of educational methods related to the characteristics of virtual reality media. Current VR programs for health care vary depending on technical or multimedia sophistication [1]. Consequently, learning through an immersive 3D VR medium using a head-mounted display (HMD) rather than a simple web-based VR system as in the case of vSim^®^ can be strongly immersive and the impact needs to be confirmed.

Therefore, this study aims to identify the effects of cognitive and emotional variables related to immersive virtual reality media technology on learning for nursing students. Through this study, it will be possible to provide insight and strategic implications for the development of learning programs and teaching–learning methods that are based on virtual reality media technology in the future.

## 2. Research Methods

### 2.1. Study Design

This study is a descriptive research study to identify the factors influencing nursing students’ learning results after experiencing virtual reality media technology.

### 2.2. Study Subjects

The subjects of this study were 121 nursing students from a university in area D who understood the purpose of this study and voluntarily agreed to participate. The number of subjects in this study was calculated using the G*Power ver. 3.1.9.7 program, which required the number of samples to be at least 109 when the median effect size was 0.15, the significance level was 0.05, the power was 0.80, and 8 other variables were calculated together based on multiple regression analysis. In consideration of the dropout rate, 121 patients were enrolled, and 120 copies (99.1%) were used as effective samples.

### 2.3. Study Parameters

#### 2.3.1. Personality Traits

Personality is composed of five sub-characteristics according to the personality theory of the Big Five Model [17]. The Big Five Index [18] was adopted by Kim and Kim [19] and a total of 22 items in a scale modified by Hong [20] were used. Each item is placed on a 6-point Likert scale, and the higher the score, the stronger the corresponding characteristic. In Hong’s study [20], Cronbach’s alpha showed Neuroticism at 0.75, Openness at 0.77, Conscientiousness at 0.75, Extraversion at 0.73, and Agreeableness at 0.66. In this study, Cronbach’s alpha was 0.82 for Neuroticism, 0.80 for Openness, 0.71 for Conscientiousness, 0.80 for Extraversion, and 0.71 for Affinity.

#### 2.3.2. Recognition of Virtual Reality Media Technology

Awareness of media technology depends on the degree of information sharing in the lecture system, the degree of support of the software, and the perception of the technical quality of the expressed information, and this was measured by modifying the tool developed by Kim [13] to be applied to the virtual reality system. This tool consists of a total of 5 items, and each item is on a 5-point Likert scale, with higher scores indicating higher awareness of media technology. In Kim’s study [13], Cronbach’s alpha was 0.52–0.64, and in this study, Cronbach’s alpha was 0.76.

#### 2.3.3. Virtual Reality Media Characteristics

As for the characteristics of the medium, the tool developed by Gye [15] for use in augmented reality-based learning systems was modified so that it could be applied to virtual reality. This tool is divided into sub-variables of sensory preoccupation and realism, and each consists of 4 items. Each item is on a 5-point Likert scale, with higher scores indicating better media characteristics. In Gye’s study [15], Cronbach’s alpha showed sensory immersion at 0.81 and realism at 0.86, and in this study, Cronbach’s alpha showed sensory immersion at 0.83 and realism at 0.83.

#### 2.3.4. Virtual Reality Media Learning Effects

A learning effect is a result of continuously maintaining the learner’s interest or motivation for learning and increasing the learner’s voluntary participation. The multimedia learning effect developed by Kim [13] was modified and used so that it could be applied to the virtual reality system. This tool consists of a total of 5 items, and each item is on a 5-point Likert scale, with higher scores indicating higher learning effects. In this study, Cronbach’s alpha was 0.81.

#### 2.3.5. Virtual Reality Learner Satisfaction

The tool for measuring the satisfaction of virtual reality learning was developed by Stein [21] and modified by Gye [15] so that it could be applied to virtual reality. It consists of a total of 4 items, and each item is on a 5-point Likert scale, with higher scores indicating higher satisfaction. In Gye’s study [15], Cronbach’s alpha was 0.80, and in this study, Cronbach’s alpha was 0.84.

#### 2.3.6. Virtual Reality Learning Necessity

The degree of thinking that virtual reality learning is necessary was measured on a visual similarity scale out of 10, which was reported to have high reliability, validity, and sensitivity when properly used as a self-reporting tool to measure subjective experiences [22].

#### 2.3.7. Intention to Continue Using Virtual Reality Learning

The degree of intention to continue using virtual reality learning was measured by a visual analog scale out of 10, which was reported to have high reliability, validity, and sensitivity when properly used as a self-reporting tool to measure subjective experiences [22].

### 2.4. Data Collection Method

After experiential learning with virtual reality from 6–8 June 2019, data was collected through questionnaires. For virtual reality learning, VIVE’s hTC VIVE ECO CE model was used. This model consists of a headset, a controller, and a base station. Experiential virtual reality learning was conducted with Head Mounted Displays (HMDs) that can select and move programs using a headset and controller. After that, only those who had written informed consent forms were asked to fill out the questionnaires. The ‘Roller Coaster’ program is a game-based virtual reality experience created for educational purposes for users who are new to virtual reality.

### 2.5. Ethical Considerations

Research participants were given an explanation about the purpose of the research, the research process, the anonymity of the research, and the guarantee of confidentiality. Surveys were conducted through a third party unrelated to the research because the researcher who conducted the experiential learning might affect the data collected directly from the research participants. The research participants were guaranteed that the collected data would be used only for research purposes, and after explaining that the collected data could be withdrawn at any time without any disadvantage, they gave their written consent.

### 2.6. Data Analysis Method

The collected data was analyzed using the IBM SPSS 26.0 program, and the analysis method is as follows.

1. The general characteristics of the subjects were analyzed using frequency, percentage, mean, and standard deviation.
2. The degree of the subject’s learning results of the virtual reality system was analyzed using descriptive statistics.
3. The difference in the learning results of the virtual reality system was analyzed by *t*-test or ANOVA according to the general characteristics of the subject and the satisfaction of the learner.
4. The relationship between the subject’s personality characteristics, their perception of virtual reality medium technology, as well as virtual reality medium characteristics, virtual reality learning effects, the learner’s satisfaction, learning necessity, and their intention to continue using virtual reality learning, were analyzed using Pearson’s correlation coefficients.
5. Multiple Regression Analysis was used to analyze the factors influencing the subject’s virtual reality learning effects.

## 3. Study Results

### 3.1. General Characteristics of the Subjects

Most subjects in this study were female (79.2%), with the average age being 24.66 years. Most were single (90.8%) and had no religion (66.7%). The majority received a monthly allowance of more than 300,000 won (67.5%). There were more cases of no part-time work (65.8%), and relatively good health was the most common (38.3%). Most answered that they had no diseases (79.2%), most of them had hobbies (85.0%), and among those hobbies, smartphone use was the most common (24.2%). Most subjects watched YouTube on a smartphone (20.8%), and the most played type of game was role-playing (5.0%). Most subjects spent 1–2 h on hobbies (17.5%) (Table 1).

### 3.2. Level of Virtual Reality Media Technology Recognition, Media Characteristics, Learning Effect, Satisfaction, Learning Necessity, and Continuous Use Intention

The average of each subject’s virtual reality technology recognition was 3.96 points, the average of the sensory immersion characteristic item in the sub-items of virtual reality medium characteristics was 3.39 points, and the average sense of realism in virtual reality mediums was 3.73 points, which were all high in the 5-point Likert scale. The average of the subjects’ virtual reality medium learning effect was 4.07 points, and the virtual reality learner satisfaction average was 4.14 points, indicating a high score on the 5-point Likert scale. The virtual reality learning necessity was 7.15 points, and the intention to continue using virtual reality learning was 7.19 points, both also indicating a high score on the similarity scale out of 10 points (Table 2).

### 3.3. Differences in Learning Effects and Learning Satisfaction According to the General Characteristics of Subjects

There was no difference in learning effect according to general characteristics of subjects, but the difference in learning satisfaction according to gender (F = 2.10, *p* = 0.038) was statistically significant (Table 3).

### 3.4. Relationship between the Learning Effects of Virtual Reality Media and the Study’s Parameters

The learning effects of the virtual reality medium had a statistically significant positive correlation with the virtual reality technology recognition, sensory immersion, realism, learning satisfaction, learning necessity, and continuous use intention (*p* < 0.001) scores. In personality traits, only Openness, Extraversion (*p* < 0.01), and Conscientiousness (*p* < 0.05) had a statistically significant positive correlation (Table 4).

### 3.5. Effects of Virtual Reality Media on Learning Effects

After the virtual reality learning experience of nursing students, to identify and understand the factors influencing the learning effects of the virtual reality medium, the learning effects as a dependent variable, along with personality characteristics, perception of virtual reality medium technology, virtual reality medium characteristics, virtual reality system learning effects, virtual reality learner’s satisfaction, virtual reality learning necessity, and intention to continue using virtual reality learning were simultaneously input as independent variables and analyzed.

In this study, the Durbin-Watson statistic was 2.009, which was close to 2, indicating that there was no autocorrelation. Then, to check the multicollinearity of the independent variables, the tolerance limit and variance expansion index (VIF) were examined. As a result, the tolerance limit value came out to 0.55–1.00, which was greater than 0.1, and the VIF was 1.00–1.81, which was less than 10, indicating that there was no multicollinearity. 

As a result of regression analysis, the explanatory power of the learning effect of the virtual reality medium was 63.9% (F = 53.61, *p* < 0.001), with learning satisfaction, sensory immersion, continuous use intention, and Extraversion being significant influencing factors (*p* < 0.05) (Table 5).

## 4. Discussion

In this study for nursing students who experienced virtual reality learning, the relationship between the characteristics of the virtual reality medium and the learner’s cognitive and emotional variables, as well as factors influencing the learning effect were identified. This study was intended to present strategic implications for the development of learning programs and teaching-learning methods using virtual reality mediums.

As a result of the study, the learning effect of virtual reality media and learner satisfaction showed high scores of 4.07 points (out of 5 points) and 4.14 points (out of 5 points). This is similar to the results of a meta-analysis study [23] that virtual reality-based learning has a great learning effect and another study [10,22] that virtual reality-based learning has a positive and significantly high educational satisfaction. A previous study [24] shows that virtual reality-based learning delivers no difference from traditional learning methods in regard to learning effects. However, considering the results of previous studies that the effect of virtual reality-based learning was high, the effect of learning can be enhanced when the traditional learning method and virtual reality-based learning are mixed and used.

However, the results of previous studies that show the effect of virtual reality-based learning was high can be seen as suggesting that the effect of learning can be improved when a mixture of the traditional learning method and virtual reality-based learning is used.

As a result of the study, the satisfaction of the learners who experienced virtual reality technology, according to the general characteristics of the subjects, was significantly higher in males than in females. This is similar to the research result in a previous study [25] that indicated that male students were more affected than female students in creative convergence classes using virtual reality production platforms. This is in line with the results of another previous study [26] in which male students were challenged and active in new learning environments, while female students preferred to learn in a stable learning environment. Therefore, when applying virtual reality technology to learning, female students may need a step-by-step approach to learning after experiencing content that can arouse their interest, such as games related to their major, along with specific usage steps for media in the pre-briefing session.

The learning effect of virtual reality had a significant positive correlation with media technology recognition, media characteristics (sensory immersion, realism), learning satisfaction, learning demand, and intention to continue learning. Although this study did not compare the effects on general learning related to using virtual reality technology, many previous studies show that virtual reality-based learning increases the learning effect overall [23,27,28,29]. On the other hand, some other previous studies also indicate that the virtual reality network method has no significant difference from the existing text-based method in learning using virtual reality media [30,31]. So, it can be observed that the virtual reality medium does not necessarily enhance the learning effect for all learning. Therefore, even if the learning effect is expected to be high due to high satisfaction with virtual reality learning, necessity, and intention to continue using it, a strategy is needed to partially use virtual reality as an auxiliary medium among all learning methods according to the type, content, and operation of learning rather than uniformly applied to learning.

Learning using virtual reality media technology is effective in knowledge acquisition, skill performance ability, collaboration ability, and educational satisfaction in several previous studies [22,32]. However, developing learning content using virtual reality to improve these abilities is a sophisticated and difficult task. This is because high-quality content development is possible only when multidisciplinary cooperation when developing learning content is required, such as the active participation of field practitioners with virtual reality technology experts together in the construction of an actual virtual reality environment. In addition, since a systematic and clear protocol for virtual reality technology-based learning can increase the learning effect, it is also a necessary task at this time to establish standard procedures and steps.

The factors influencing the learning results after the virtual reality learning experience of nursing students were learning satisfaction, sensory immersion from the media characteristics, intention to continue learning, and extroversion from the personality characteristics. The influence of these factors was very high at 63.9%. Previous studies also showed that the satisfaction of learning in the virtual reality medium and the sensory immersion in learning had a significant influence on the learning effect [25,33,34]. This indicates that learning using virtual reality technology can have different learning effects depending on the type, content, and operation method of the learning as well as the learner’s cognitive and emotional variables, and it is necessary to consider the learner’s cognitive and emotional variables. Also, among virtual reality media technologies, immersive 3D virtual reality technology is known to be superior to simple web-based virtual reality in regard to immersion in learning [35]. Therefore, the active use of virtual reality learning may be required, but the use of immersive 3D virtual reality mediums that can increase the learning effect has not yet been systematized, and the development of learning content is minimal. In the future, as the overall technology for immersive 3D virtual reality mediums and the areas related to the development of learning content are expected to continue developing, we hope that research on learning effects, effective learning types, contents, and methods will be actively conducted in line with this.

In this study, subjects reported experiencing the discomfort of wearing equipment (which was more severe when wearing glasses), physical discomfort (headache, dizziness, fatigue), and other problems (lack of content, lack of equipment proficiency, and a lack of available equipment due to high expense). Therefore, when developing immersive 3D virtual reality technology-based learning content in the future, technical and physical disadvantages such as wearing glasses and complaining of physical discomfort should be improved together. When applied to learning, countermeasures to deal with students who complain of physical discomfort should be prioritized.

As the subjects of this study were some students from a single university, there may be limitations in generalizing the research results. In addition, due to the diversity of virtual reality media in virtual reality-based learning, the immersive 3D virtual reality media used in this study may include economic and environmental difficulties such as equipment installation and program installation, so an overall comparative analysis of virtual reality-based learning was conducted.

## 5. Conclusions

This study attempted to identify factors influencing the effect on learning when using virtual reality media technology for nursing students, and to provide basic data for developing and applying virtual reality media technology-based learning programs in the future. 

The significance of this study is that it provides the insight necessary to develop a learning program using virtual reality technology and includes strategic implications in the teaching and learning method of virtual reality technology-based learning while considering the characteristics of virtual reality technology and the cognitive and psychological variables of learners.

In future learner education: (1) When developing a learning program using virtual reality media technology, it is necessary to consider the type and content of learning, operation method, and cognitive and emotional variables of learners; and (2) Rather than applying the method uniformly to all learning, it is necessary to maximize the effect of virtual reality learning by partially utilizing it as an auxiliary medium for related learning.

Future research suggests that: (1) Repeated studies should be conducted on the differences in learning effects according to the type and content of learning, the operation method, the variables of the learner, and the type of virtual reality medium; and (2) It is also suggested that active research be conducted in the future on interventions and countermeasures against physical discomfort that may occur during virtual reality-based learning.

## Figures and Tables

**Table 1:** General Characteristics of Subjects.

Characteristics	Categories	N (%)	Mean(SD)
Gender	Male	25 (20.8)	
	Female	95 (79.2)	
Age (/year)	≤21	8 (6.7)	24.66 (5.71)
	22–23	68 (56.7)	
	24–25	22 (18.3)	
	≥26	22 (18.3)	
Marital status	No	109 (90.8)	
	Yes	11 (9.2)	
Religion	No	80 (66.7)	
	Yes	40 (33.3)	
Pocket money (/month)	<100,000 won	2 (1.7)	
	10–190,000 won	8 (6.7)	
	20–290,000 won	29 (24.2)	
	≥300,000 won	81 (67.5)	
Arbeit	No	79 (65.8)	
	Yes	41 (34.2)	
Subjective health	Very good	1 (0.8)	
	Good	19 (15.8)	
	Moderate	46 (38.3)	
	Bad	43 (35.8)	
	Very bad	11 (9.2)	
Disease	No disease	95 (79.2)	
	physical disease	22 (18.3)	
	mental disease	2 (1.7)	
	physical & mental disease	1 (0.8)	
Hobby	No	18 (15.0)	
	Yes	102 (85.0)	
	smartphone	29 (24.2)	
	game	19 (15.8)	
	listening to music	14 (11.7)	
	watching movie	11 (9.2)	
	others	47 (39.1)	
Smartphone	YouTube	25 (20.8)	
	chatting	9 (7.5)	
	searching	5 (4.2)	
	others	81 (67.5)	
Game	role playing	6 (5.0)	
	arcade	5 (4.2)	
	simulation	4 (3.3)	
	others	105 (87.5)	
Time spent on hobby	≤1 h	18 (15.0)	
	1–2 h	21 (17.5)	
	2–3 h	13 (10.8)	
	3–4 h	12 (10.0)	
	>4 h	10 (8.3)	
	others	46 (38.4)	

**Table 2:** Level of virtual reality media technology recognition, media characteristics, learning effect, satisfaction, learning necessity, and continuous use intention.

Variables		Range	Min	Max	M ± SD	Item M ± SD
Virtual reality media technology recognition		5–25	13.00	25.00	19.82 ± 2.52	3.96 ± 0.51
Virtual reality media characteristics	Sensory immersion	4–20	4.00	20.00	13.54 ± 2.90	3.39 ± 0.73
	Realism	4–20	4.00	20.00	14.93 ± 2.95	3.73 ± 0.74
Learning effect		5–25	12.00	25.00	20.34 ± 2.96	4.07 ± 0.59
Learning satisfaction		4–20	9.00	20.00	16.57 ± 2.48	4.14 ± 0.62
Learning necessity		0–10	2.00	10.00	7.15 ± 1.66	
Intention to use continuously		0–10	1.00	10.00	7.19 ± 1.77	

**Table 3:** Differences in learning effect and learning satisfaction according to the general characteristics of subjects.

Characteristics	Categories	Learning Effect		Learning Satisfaction	
		Mean ± SD	t or F(*p*)	Mean ± SD	t or F(*p*)
Gender	Male	21.32 ± 2.81	1.88 (0.063)	17.48 ± 2.43	2.10 (0.038 *)
	Female	20.08 ± 2.96		16.33 ± 2.45	
Age (/year)	≤21	20.13 ± 3.48	1.77 (0.156)	16.50 ± 2.78	1.98 (0.122)
	22–23	19.94 ± 3.05		16.22 ± 2.60	
	24–25	21.59 ± 2.79		17.68 ± 1.96	
	≥26	20.41 ± 2.44		16.55 ± 2.26	
Marital status	No	20.31 ± 2.96	−0.35 (0.731)	16.51 ± 2.51	−0.73 (0.464)
	Yes	20.64 ± 3.11		17.09 ± 2.17	
Religion	No	20.14 ± 2.97	−1.07 (0.287)	16.44 ± 2.44	−0.81 (0.422)
	Yes	20.75 ± 2.93		16.83 ± 2.56	
Pocket money (/month)	<100,000 won	22.50 ± 3.54	2.02 (0.116)	17.00 ± 1.41	0.94 (0.423)
	10–190,000 won	18.13 ± 4.09		15.25 ± 3.50	
	20–290,000 won	20.28 ± 2.23		16.90 ± 2.21	
	≥300,000 won	20.53 ± 3.01		16.57 ± 2.48	
Arbeit	No	20.40 ± 2.84	0.35 (0.726)	16.50 ± 2.51	−0.38 (0.705)
	Yes	20.20 ± 3.23		16.68 ± 2.46	
Subjective health	Very good	23.00 ± 0.00	0.24 (0.916)	20.00 ± 0.00	0.92 (0.456)
	Good	20.16 ± 2.67		15.95 ± 2.66	
	Moderate	20.26 ± 3.12		16.48 ± 2.07	
	Bad	20.40 ± 2.69		16.84 ± 2.52	
	Very bad	20.55 ± 4.03		16.64 ± 3.50	
Disease	No disease	20.23 ± 3.02	0.66 (0.576)	16.55 ± 2.46	1.32 (0.272)
	physical disease	21.00 ± 2.85		16.91 ± 2.56	
	mental disease	19.50 ± 0.71		16.00 ± 0.00	
	physical & mental disease	18.00 ± 0.00		12.00± 0.00	
Smartphone	YouTube	20.80 ± 3.56	0.78 (0.510)	16.76 ± 3.22	0.63 (0.599)
	chatting	20.33 ± 2.78		16.22 ± 1.72	
	searching	18.60 ± 2.19		15.20 ± 1.79	
	others	20.31 ± 2.82		16.63 ± 2.33	

Note: * *p* < 0.05.

**Table 4:** Relationship between the learning effects of virtual reality media and the study’s parameters.

Variables		r (*p*)						
		(1)	(2)	(3)	(4)	(5)	(6)	(7)
Media technology recognition ^(1)^		1						
Media Characteristics	Sensory Immersion ^(2)^	0.29 (0.001)	1					
	Realism ^(3)^	0.30 (0.001)	0.64 (<0.001)	1				
Learning Effect ^(4)^		0.56 (<0.001)	0.34 (<0.001)	0.35 (<0.001)	1			
Learning Satisfaction ^(5)^		0.58 (<0.001)	0.23 (0.011)	0.34 (<0.001)	0.77 (<0.001)	1		
Learning Necessity ^(6)^		0.48 (<0.001)	0.27 (0.002)	0.29 (0.001)	0.56 (<0.001)	0.57 (<0.001)	1	
Intention to use continuously ^(7)^		0.46 (<0.001)	0.19 (0.040)	0.25 (0.007)	0.60 (<0.001)	0.65 (<0.001)	0.67 (<0.001)	1
Personality traits	Neuroticism	−0.10 (0.298)	−0.00 (0.980)	−0.11 (0.230)	−0.00 (0.978)	−0.06 (0.524)	−0.11 (0.248)	−0.15 (0.095)
	Openness	0.16 (<0.083)	0.10 (0.302)	0.08 (0.418)	0.25 (0.006)	0.26 (0.005)	0.19 (0.035)	0.25 (0.007)
	Conscientiousness	0.18 (0.046)	0.12 (0.197)	0.02 (0.827)	0.20 (0.027)	0.17 (0.063)	0.20 (0.027)	0.24 (0.008)
	Extraversion	0.15 (0.115)	0.01 (0.900)	−0.06 (0.537)	0.28 (0.002)	0.21 (0.019)	0.05 (0.619)	0.15 (0.094)
	Affinity	0.10 (0.256)	0.01 (0.938)	−0.03 (0.756)	0.00 (0.984)	0.09 (0.352)	−0.01 (0.894)	−0.01 (0.908)

**Table 5:** Factors Influencing the Learning Effects of Virtual Reality Media.

Variables	B	SE	β	t	*p*
(Constant)	2.008	1.423		1.411	0.161
Learning Satisfaction	0.701	0.089	0.587	7.913	0.000
Sensory Immersion	0.195	0.058	0.191	3.362	0.001
Intention to use continuously	0.274	0.121	0.164	2.260	0.026
Extraversion	0.125	0.056	0.127	2.245	0.027

Adj.R^2^ = 63.9, F = 53.607, *p* < 0.001.

## Data Availability

Not available.
